# Embryonic myosin is a regeneration marker to monitor utrophin-based therapies for DMD

**DOI:** 10.1093/hmg/ddy353

**Published:** 2018-10-09

**Authors:** Simon Guiraud, Benjamin Edwards, Sarah E Squire, Lee Moir, Adam Berg, Arran Babbs, Nesrine Ramadan, Matthew J Wood, Kay E Davies

**Affiliations:** Department of Physiology, Anatomy and Genetics, MDUK Oxford Neuromuscular Centre, University of Oxford, Oxford OX1 3PT, United Kingdom

## Abstract

Duchenne muscular dystrophy (DMD) is a lethal, X-linked muscle-wasting disease caused by lack of the cytoskeletal protein dystrophin. Constitutive utrophin expression, a structural and functional paralogue of dystrophin, can successfully prevent the dystrophic pathology in the dystrophin-deficient *mdx* mouse model. In dystrophic muscles, utrophin is increased as part of the repair process and localized at the sarcolemma of regenerating myofibers. The presence of developmental myosin such as embryonic myosin (MyHC-emb) and neonatal represents a useful marker of muscle regeneration and a meaningful indicator of muscle damage, which correlates with the clinical severity of milder Becker muscular dystrophy and DMD patients. In the present study, we demonstrate that MyHC-emb is a robust marker of regeneration at different ages and in different skeletal muscles. We also evaluate the correlation between utrophin, dystrophin and MyHC-emb in wild-type (wt) and regenerating dystrophic muscles. Restoration of dystrophin significantly reduced MyHC-emb levels. Similarly, overexpression of utrophin in the transgenic *mdx*-Fiona mice reduced the number of MyHC-emb positive fibers to wt level, prevented the regenerative process and rescued the muscle function. In contrast, the absence of utrophin in the dystrophin-deficient double-knockout mice resulted in a higher MyHC-emb content and in a more severe dystrophic pathophysiology than in *mdx* mice. These data illustrate the importance of monitoring utrophin and MyHC-emb levels in the preclinical evaluation of therapies and provide translational support for the use of developmental myosin as a disease biomarker in DMD clinical trials.

## Introduction

Duchenne muscular dystrophy (DMD) is a fatal X-linked recessive neuromuscular disease affecting 1 in 5000 newborn males (1,2). This disorder is caused by mutations in the DMD gene (OMIM 300377, Xp21.2-p21.1) (3,4), presenting one of the highest rates in new mutations, which are predominantly deletions of the gene (5). The DMD gene encodes for dystrophin (Uniprot P11532), an essential 427 kDa cytoplasmic protein that establishes a mechanical link between the extracellular matrix and the actin cytoskeleton through the dystrophin-associated protein complex (DAPC) (6). Dystrophin is critical for the maintenance of the biomechanical properties of fiber strength, flexibility and stability in skeletal muscle, allowing myofibers to cope with repeated cycles of muscle contraction and relaxation (7). The absence or reduction of dystrophin in the milder Becker muscular dystrophy (BMD, MIM #300376) (8) leads to sarcolemma fragility and subsequent chronic inflammation associated with repeated cycles of muscle necrosis and regeneration. Adipose and connective tissue progressively replace muscle fibers and lead to reduction in muscle mass and function, and ultimately to loss of muscle fibers (1). DMD patients manifest the first onset of symptoms such as walking abnormalities, abnormal gait, proximal muscle weakness and calf muscle pseudo-hypertrophy in their early infancy. These symptoms progress relentlessly to loss of ambulation generally by the age of 12 years (9) and patients develop respiratory and cardiac failure leading to premature death by their early 30s (10).

Despite exhaustive clinical management of cardiac complications, assisted ventilation and corticosteroid treatment (11,12), there is presently no cure for DMD. The urgency to seek an effective treatment for DMD has resulted in the development of genetic and pharmacological interventions to correct or compensate for dystrophin deficiency, such as exon skipping (13,14), stop codon readthrough (15) and dystrophin gene therapies (16). Approaches to mitigate secondary and downstream pathological mechanisms (17,18) in parallel with translational efforts to define more accurate biomarkers and endpoints (19,20) have been also undertaken. Over the past two decades, more than 200 clinical trials in DMD patients have been conducted, are ongoing or are recruiting. To date, none have shown clear significant clinical efficacy, but recently microdystrophin gene therapy has shown promising interim results in the phase 1/2 trials (21).

Utrophin is a structural and functional autosomal paralogue of dystrophin (22,23). Both utrophin and dystrophin have structurally similar N-terminal, cysteine-rich and C-terminal domains (24,25) and share many binding partners, such as β-dystroglycan, α-dystrobrevin-1 and F-actin (25). Utrophin and dystrophin differ by their spatio-temporal expression. In developing muscles, utrophin (26,27) is expressed at the sarcolemma and is progressively replaced by dystrophin (28). In adult tissues, utrophin is expressed in a wide range of tissues such as lung, kidney, liver and spleen (29) with the utrophin-A isoform confined to the neuromuscular (NMJ) and myotendinous junctions (30) and the sarcolemma in regenerating myofibers (31). Utrophin-B is limited to blood vessels (29). Despite subtle differences, such as recruitment of the neuronal nitrogen synthase (32), the mode of interaction with microtubules (33) and the F-actin filaments (34), the high level of structural identity and relative conservation led to the hypothesis that utrophin might be an effective surrogate to compensate for the lack of dystrophin in dystrophic muscles (35). The generation of transgenic *mdx* mice established that ubiquitous over-expression of full-length utrophin, and its continuous localization along the muscle membrane suppresses histophysiological signs of dystrophinopathy in a dose dependent manner (36–38) with no detrimental effect (39). High levels of utrophin (3–5×) observed in the transgenic *mdx*-Fiona mouse offer significant functional rescue of the dystrophic phenotype and correct a large majority of serological *mdx* biomarkers (40). Uniform expression, even at low levels of utrophin (1.5× compared to *mdx*) can be beneficial (36). The significant advantage of utrophin modulation therapy is that the approach is applicable to all DMD patients, regardless of the dystrophin mutation.

The long-term therapeutic aim is to develop a systemic universal therapy by maintaining utrophin levels at the muscle membrane to compensate for the lack of dystrophin in patients. Alongside direct delivery of the protein (41), stabilization of the protein or RNA (42,43), viral approaches (44,45) and non-viral strategies such as recombinant biglycan (46), we have shown that utrophin modulators improve muscle function in *mdx* mice (47). Increased utrophin levels after small drug administration improved sarcolemmal stability and resulted in a significant reduction in the key hallmarks of the disease such as regeneration, necrosis and fibrosis, which translated into physiological and muscle function improvements in *mdx* mice.

In mouse and human dystrophic muscles, utrophin is increased by 2–5-fold over wild type (wt) as part of the repair process and localized at the sarcolemma of small regenerating fibers (31,48). However, the levels of regeneration and utrophin vary between muscle types and are age-dependent in both mice and DMD boys, emphasizing the complexity of quantifying utrophin in dynamic and dystrophic muscles (22). As utrophin is a regeneration-associated protein increased in dystrophic muscles (49), it is essential to correlate the utrophin levels after drug treatment with regeneration markers. During muscle development, skeletal muscle expresses myosin isoforms such as embryonic (MyHC-emb) and neonatal myosin heavy chains, respectively encoded by the *myh3* and *myh8* genes (50). These specific cytoskeletal motor proteins are transiently expressed during embryonic and fetal development and disappear after birth when adult slow and fast myosins become prevalent (51). After injury or in neuromuscular disorders such as DMD, developmental isoforms of myosin are re-expressed (52) during muscle regeneration and are detected in newly formed regenerating myofibers 2–3 days after injury and persist for 2–3 weeks (50). Numerous studies have reported elevated developmental myosin levels in *mdx* mice (53), in the canine X-linked muscular dystrophy (54) and in BMD and DMD patients (52,55,56). Thus, the presence of these embryonic and neonatal cytoskeletal motor proteins represents a useful marker of muscle regeneration and a meaningful indicator of muscle damage, as levels of developmental myosin correlate with the clinical severity of Becker and DMD patients (55).

In the present study, we evaluated, in detail, the correlation between utrophin/dystrophin and MyHC-emb levels in wt and regenerating *mdx* muscles at different ages and different muscles. Our data provide translational support for use of developmental myosin as a disease biomarker in DMD clinical trials.

## Results

### Embryonic myosin expression in dystrophic muscles

**Figure 1:**
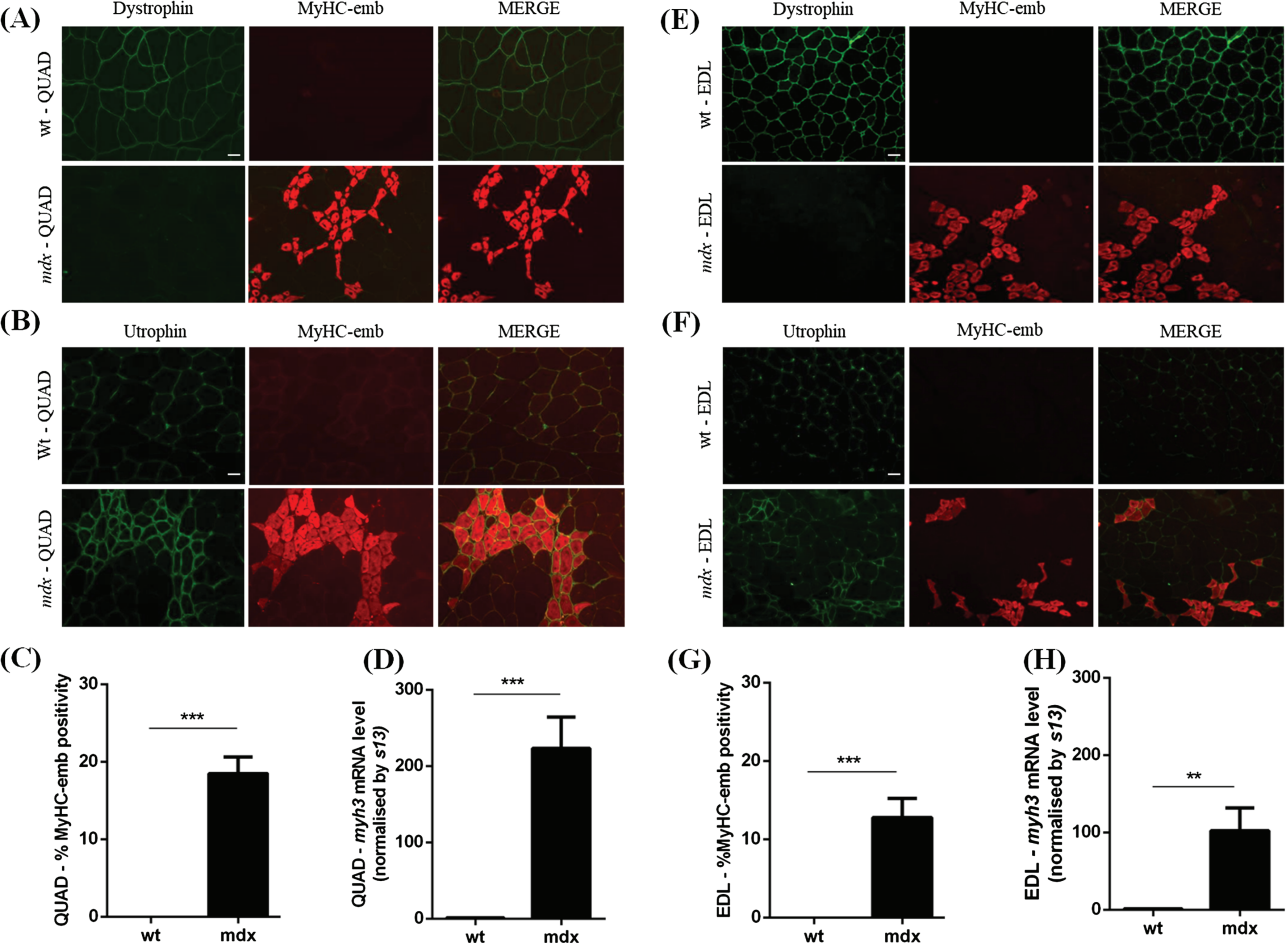
Embryonic myosin levels in 7-week-old wt and *mdx* skeletal muscles. **(A)** Co-immunofluorescence staining for dystrophin/MyHC-emb, in 7-week-old wt and *mdx* QUAD muscles. Dystrophin is expressed at the muscle membrane in wt and absent in *mdx* tissue. MyHC-emb is negligible in wt and increased in *mdx* muscle. Scale bar: 100 μm. **(B)** Co-immunofluorescence staining for utrophin/MyHC-emb, in 7-week-old wt and *mdx* QUAD muscles. Utrophin is limited to the NMJ and blood vessels in wt and increased at the sarcolemma of regenerating *mdx* myofibers defined by the expression of MyHC-emb. Scale bar: 100 μm. **(C)** MyHC-emb positive myofibers in wt and *mdx* 7-week-old QUAD sections. In *mdx* QUAD muscle, 18.5% myofibers are positive for MyHC-emb. **(D)***myh3* mRNA normalized with *13 s* in 7-week-old wt and *mdx* QUAD. A significant 223-fold increase of the *myh3* transcripts was noted in *mdx* muscle compared to wt. **(E)** Co-immunofluorescence staining for dystrophin/MyHC-emb, in 7-week-old wt and *mdx* EDL muscles. Expression pattern as described in QUAD. Scale bar: 100 μm. **(F)** Co-immunofluorescence staining for utrophin/MyHC-emb, in 7-week-old wt and *mdx* EDL muscles. Expression pattern as described in QUAD. Scale bar: 100 μm. **(G)** MyHC-emb positive myofibers in wt and *mdx* 7-week-old EDL sections. In *mdx* EDL muscle, 12.8% myofibers are positive for MyHC-emb. **(H)***myh3* mRNA normalized with 13 s in 7-week-old wt and *mdx* EDL. A significant 102-fold increase of the *myh3* transcripts was noted in *mdx* muscle compared to wt. Values are mean ± SEM of *n* = 6 per condition; ^*^P < 0.05, ^**^P < 0.01, ^***^P < 0.01.

We evaluated the expression of MyHC-emb, a marker of regeneration, in 7- and 14-week-old quadriceps (QUAD) and extensor digitorum longus (EDL) muscles in both wt C57BL/10 and dystrophin-deficient *mdx* mice (57). In 7-week-old wt skeletal muscles, dystrophin is localized at the muscle membrane (Fig. 1A and E), and utrophin is confined to the NMJ and blood vessels (Fig. 1B and F). In *mdx* muscle, dystrophin is absent (Fig. 1A, E) and utrophin is inconsistently increased at the sarcolemma (Fig. 1B, F). Between 3 and 5 weeks of age, *mdx* muscle undergoes a temporary major DMD-like crisis with muscle fiber degeneration and necrosis, followed by extensive compensatory myofiber regenerative and repair processes until 12 weeks of age (58,59). At 7 weeks of age, MyHC-emb is increased in *mdx* muscles as a marker of regeneration (Fig. 1A–F). In *mdx* muscle, increased utrophin is predominantly co-localized with MyHC-emb and expressed in regenerative fibers (Fig. 1B, F). Using merosin as a mask and a co-laminin-α2/MyHC-emb immunofluorescence stain (Supplementary Material, Fig S1 A–D), we determined the percentage of MyHC-emb positive fibers in wt and *mdx* skeletal muscles. In 7-week-old QUAD and EDL muscles, wt mice show negligible MyHC-emb expression, while *mdx* mice show 18.5 and 12.8% MyHC-emb positive fibers, respectively (Fig. 1C and G). Consistently, at 7 weeks of age, the transcripts of *myh3* were significantly increased by 223-fold in *mdx* QUAD and by 102-fold in *mdx* EDL muscles compared to wt (Fig. 1D, H). Both *mdx* skeletal muscles present a highly significant increase of MyHC-emb level at 7 weeks of age compared to wt muscle, but interestingly, the QUAD muscle showed more regeneration than the EDL muscle. Consistent with these results, we observed a higher macrophage (F4/80) infiltration in QUAD muscle (Supplementary Material, Fig. S2).

**Figure 2:**
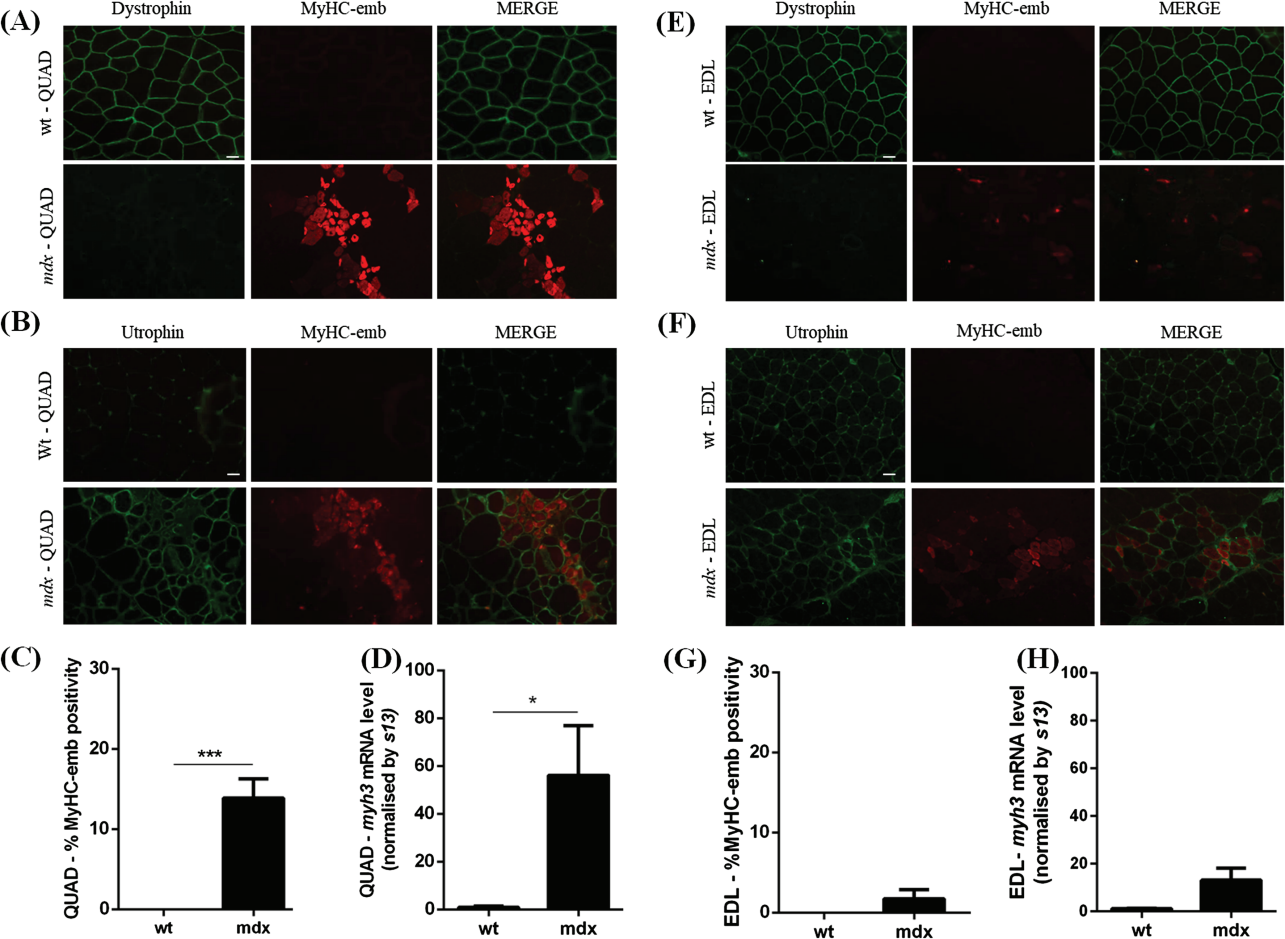
Embryonic myosin levels in 14-week-old wt and *mdx* skeletal muscles. **(A)** Co-immunofluorescence staining for dystrophin/MyHC-emb in 14-week-old wt and *mdx* QUAD muscles. Dystrophin is expressed at the sarcolemma in wt and absent in *mdx* muscle. MyHC-emb is negligible in wt and increased in *mdx* tissue. Scale bar: 100 μm. **(B)** Co-immunofluorescence staining for utrophin/MyHC-emb in 14-week-old wt and *mdx* QUAD muscles. Utrophin is confined to the NMJ and blood vessels in wt and increased at the muscle membrane of regenerating *mdx* myofibers. MyHC-emb signal increased in *mdx* muscle and allows tracking utrophin associated with regenerative process. Scale bar: 100 μm. **(C)** MyHC-emb positive myofibers in wt and *mdx* 14-week-old QUAD sections. In *mdx* QUAD muscle, 13.9% myofibers are positive for MyHC-emb. **(D)***myh3* mRNA normalized with *13 s* in 14-week-old wt and *mdx* QUAD. A significant 56-fold increase of the *myh3* transcripts was noted in *mdx* QUAD muscle compared to wt. **(E)** Co-immunofluorescence staining for dystrophin/MyHC-emb, in 14-week-old wt and *mdx* EDL muscles. Dystrophin is expressed at the sarcolemma in wt and absent in *mdx* muscle. MyHC-emb is negligible in wt and low in *mdx* tissue. Scale bar: 100 μm. **(F)** Co-immunofluorescence staining for utrophin/MyHC-emb, in 14-week-old wt and *mdx* EDL muscles. Scale bar: 100 μm. **(G)** MyHC-emb positive myofibers in wt and *mdx* 14-week-old EDL. In *mdx* EDL muscle, 1.7% myofibers are positive for MyHC-emb. **(H)***myh3* mRNA normalized with 13 s in 14-week-old wt and *mdx* EDL. No significant increase in the *myh3* transcripts was noted in *mdx* 14-week-old EDL muscle compared to wt. Values are mean ± SEM of *n* = 6 per condition; ^*^P < 0.05, ^**^P < 0.01, ^***^P < 0.01.

**Figure 3:**
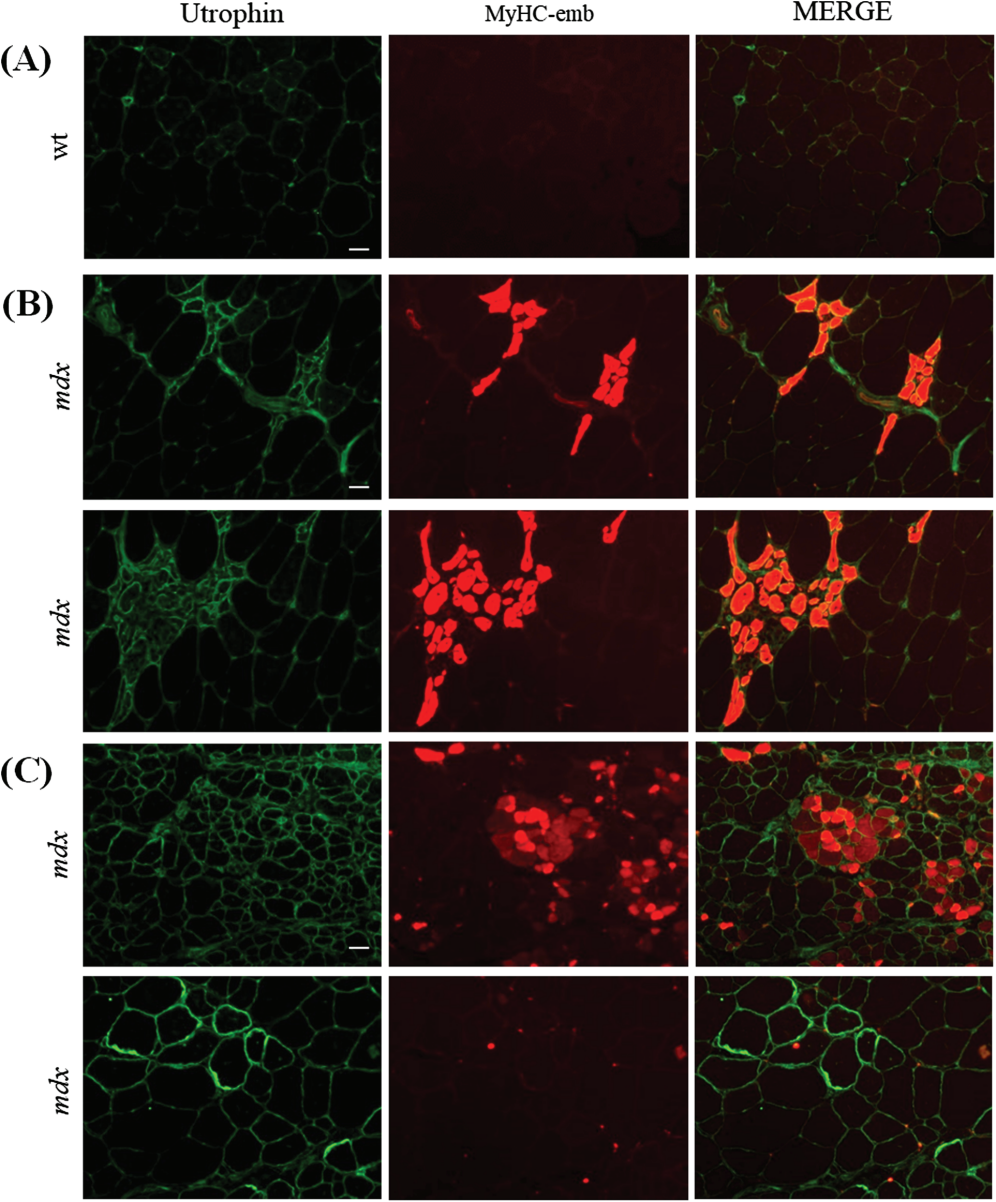
Embryonic myosin signal allows to track only regeneration-associated utrophin signal. **(A)** Utrophin/MyHC-emb co-immunofluorescence staining in transverse section of 14-week-old wt QUAD. Scale bar: 100 μm. **(B)** Utrophin/MyHC-emb co-immunofluorescence staining in transverse section of 14-week-old *mdx* QUAD showing that utrophin is increased in regenerating fiber positive for MyHC-emb. Scale bar: 100 μm. **(C)** Utrophin/MyHC-emb co-immunofluorescence staining in transverse section of 14-week-old *mdx* QUAD showing that utrophin is increased in non-regenerating fibers negative for MyHC-emb. Scale bar: 100 μm.

Following the peak of regeneration between the ages of 6 and 12 weeks (60), *mdx* muscle enters a slight and chronic dystrophic phase, which does not recapitulate the human pathology. In correlation with published conclusions, 14-week-old dystrophin deficient-*mdx* QUAD muscles contain 13.9% MyHC-emb positive fibers (Fig. 2A–C) and show 56-fold increase *myh3* mRNA expression (Fig. 2D). Despite lower levels of MyHC-emb, reflecting a lower level of regeneration at 14 weeks of age (when compared to 7 weeks), levels are still significantly increased in 14-week-old *mdx* QUAD compared to wt QUAD (Fig. 2A–D). In 14-week *mdx* EDL, MyHC-emb signal (Fig. 2E–G) and *myh3* levels (Fig. 2H) are not significantly different from wt EDL, suggesting that the regeneration process is specific to the age. Consequently, MyHC-emb may be used as marker of regeneration only in the appropriate regenerative context. It is important to note that MyHC-emb correlates with regeneration-associated utrophin and may not offer a complete representation of the total utrophin signal across all the fibers as utrophin is also expressed at the sarcolemma of some myofibers that are MyHC-emb negative (Fig. 3B, C). This observation may be explained by different regeneration states between muscle fibers, the limitation of the transiently MyHC-emb signal persistent for 2–3 weeks in dystrophic muscle, an increase of utrophin in dystrophic tissue independently from the regenerative process and the stabilization of the utrophin protein at the DPAC.

As previously demonstrated by Roma and colleagues (61), developmental myosin protein was detected in western blots within the first 5 days of life in wt and *mdx* animals. Therefore, as expected, MyHC-emb protein was not detected by western blot in our 7- and 14-week-old samples (Supplementary Material, Fig. S3). We also studied mRNA expression levels of *myh8* in 7-week-old QUAD and observed a 54.4-fold increase in *mdx* tissue (Supplementary Material, Fig. S4) compared to the 223-fold increase observed of *myh3* mRNA levels (Fig. 1D). Therefore, we focused our study only on MyHC-emb.

Taken together, these results indicate that MyHC-emb, detected by imaging and mRNA quantitation, is a robust marker of regeneration in 7-week-old *mdx* QUAD and EDL and in 14-week *mdx* QUAD muscles.

### Dystrophin restoration reduces MyHC-emb levels and regeneration

To investigate the benefits of dystrophin restoration on regeneration and MyHC-emb levels, we treated 12-week-old *mdx* animals with Pip9b2-PMO, an arginine-rich cell-penetrating peptide conjugated to a phosphorodiamidate morpholino oligonucleotide (62). This dystrophin-based strategy modulates dystrophin pre-mRNA splicing by restoring the reading frame of the murine dystrophin gene via exon 23 skipping and therefore generates a truncated but semi-functional dystrophin protein isoform.

**Figure 4:**
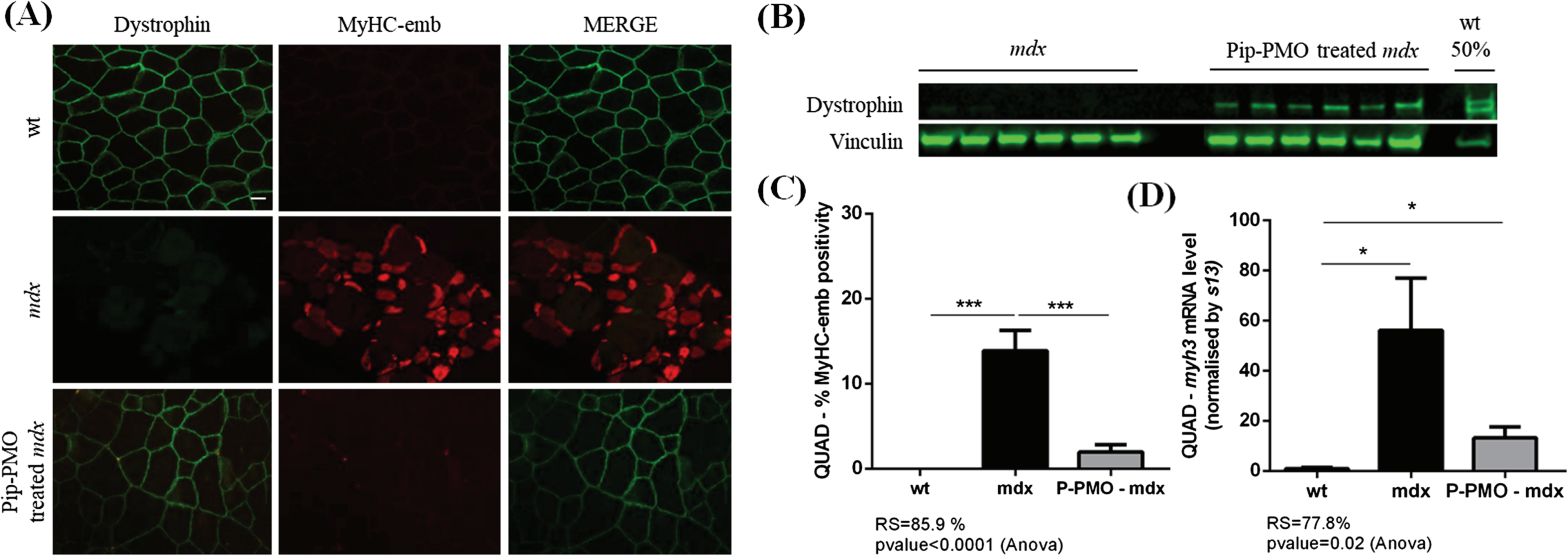
Restoration of dystrophin expression with Pip-PMO rescues embryonic myosin levels. **(A)** Co-immunofluorescence staining for dystrophin/MyHC-emb, in 14-week-old wt, *mdx* and Pip-PMO treated *mdx* QUAD. In wt QUAD, dystrophin is localized at the muscle membrane and MyHC-emb signal is negligible; in *mdx* muscle, dystrophin is absent and MyHC-emb signal increased; after Pip-PMO treatment, dystrophin is partially rescued at the sarcolemma and MyHC-emb reduced toward wt levels. Scale bar: 100 μm. **(B)** Relative dystrophin protein levels in untreated *mdx*, Pip-PMO treated *mdx* and wt 14-week QUAD detected via western blot. Vinculin was used to control for equal loading and to normalize dystrophin protein expression. **(C)** MyHC-emb positive myofibers in wt, *mdx* and Pip-PMO treated *mdx* QUAD. In 14-week-old *mdx* QUAD, 13.9% myofibers are positive for MyHC-emb and dystrophin restoration significantly reduces MyHC-emb toward wt level with an 85.9% recovery score. **(D)***myh3* mRNA normalized with 13 s in wt, *mdx* and Pip-PMO-treated *mdx* QUAD. *mdx* QUAD presents a significant 56-fold increase of the *myh3* transcripts compared to wt. Dystrophin rescued by Pip-PMO reduces *myh3* mRNA level with a 77.8% recovery score. Values are mean ± SEM of n = 6 per condition; ^*^P < 0.05, ^**^P < 0.01, ^***^P < 0.01.

After a single intravenous 10 mg/kg dose, a 2-week-old duration treatment with Pip-PMO; previously described as efficient for moderating the pathology (63) results in a 20% restoration of wt dystrophin levels (Fig. 4B). In treated animals, a robust dystrophin signal was localized at the muscle membrane of QUAD muscle (Fig. 4A). The rescued dystrophin reduced the MyHC-emb signal [recovery score (RS) = 85.9%; Fig. 4C, Supplementary Material, Fig. S4A] and the *myh3* mRNA level (RS = 77.8%; Fig. 3D) toward wt level, demonstrating a diminution in regeneration. Thus, reduced MyHC-emb levels are linked to dystrophin restoration and resulting increased membrane stability.

### Embryonic myosin levels in different utrophin contexts

**Figure 5:**
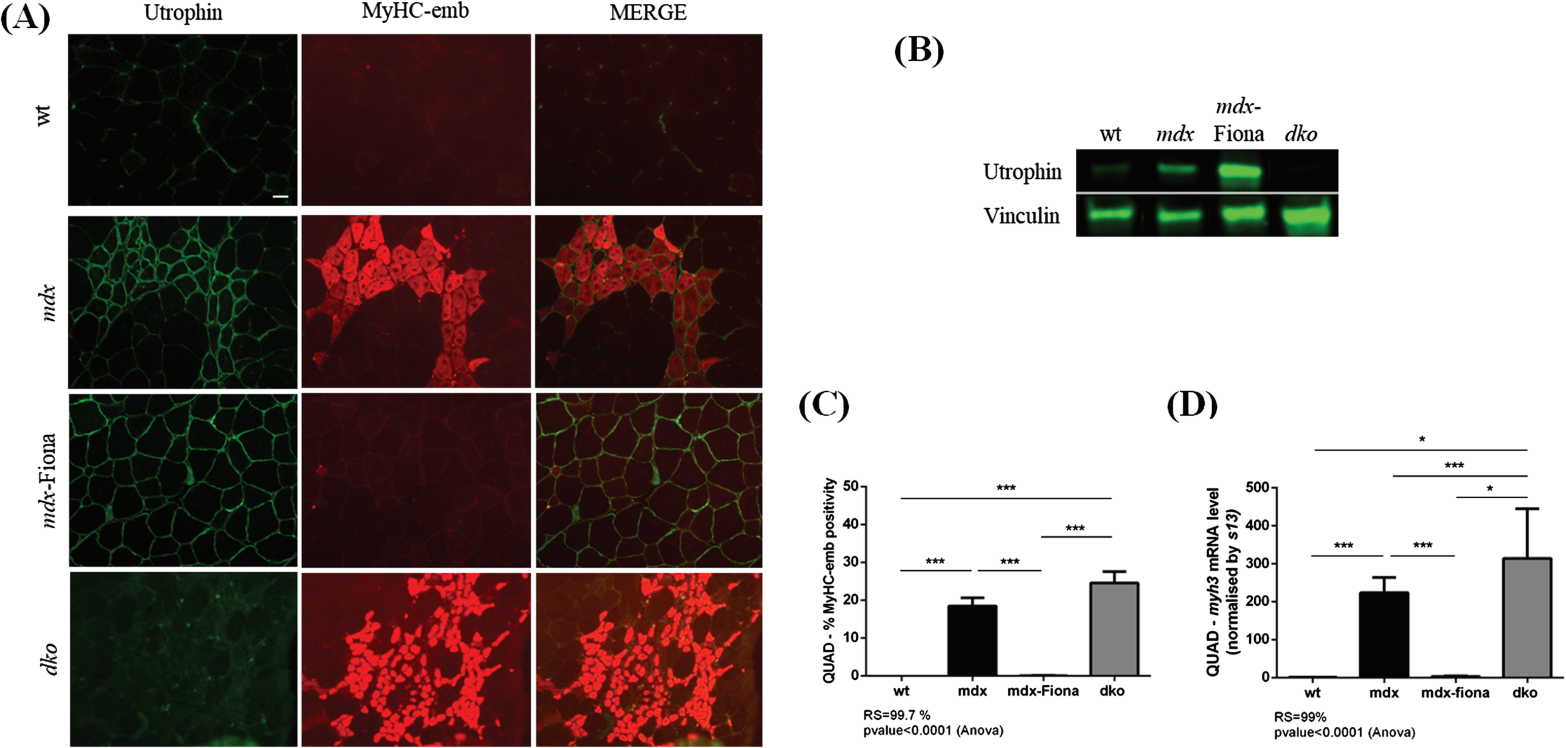
Utrophin contexts influence embryonic myosin levels in 7-week-old QUAD muscles. **(A)** Co-immunofluorescence staining for utrophin/MyHC-emb, in 7-week-old wt, *mdx*, *mdx*-Fiona and *dko* QUAD. In wt QUAD, utrophin is limited to the NMJ and blood vessels and MyHC-emb is negligible. In *mdx* tissue, MyHC-emb signal is increased reflecting a higher level of regeneration and utrophin is increased and localized at the muscle membrane of regenerating fibers. In *mdx*-Fiona, utrophin is expressed at high levels and consistently localized at the sarcolemma; MyHC-emb is negligible. In *dko*, utrophin is absent, the dystrophic phenotype worsens and MyHC-emb increased compared to *mdx* tissue. Scale bar: 100 μm. **(B)** Representative relative utrophin protein levels in wt, *mdx*, *mdx*-Fiona and *dko* 7-week QUAD detected via western blot. Utrophin is increased by 2.5-fold in *mdx* compared to wt; *mdx*-Fiona present a 3-fold increase of total utrophin protein compared to *mdx* and utrophin is absent in *dko*. Vinculin was used to control for equal loading and to normalize utrophin protein expression. **(C)** MyHC-emb positive myofiber in wt, *mdx*, *mdx*-Fiona and *dko* QUAD. In *mdx* QUAD, 18.5% myofibers are positive for MyHC-emb and high level of utrophin in *mdx*-Fiona fully rescue MyHC-emb to wt levels with a 99.7% recovery score. There is a higher number of MyHC-emb positive myofibers in *dko* tissue compared to *mdx*. **(D)***myh3* mRNA normalized with *13s* in wt, *mdx*, *mdx*-Fiona and *dko* QUAD. The *myh3* transcripts are fully rescued to wt levels in the utrophin overexpressing *mdx-*Fiona mice. Absence of utrophin in the dystrophin-deficient *dko* results in a significant higher *myh3* mRNA levels compared to *mdx*. Values are mean ± SEM of n = 6; ^*^P < 0.05, ^**^P < 0.01, ^***^P < 0.01.

Similarly to dystrophin, overexpression of utrophin can improve the membrane stability of dystrophic myofibers and suppress the functional signs of dystrophinopathy (36,37). In order to assess positive as well as negative outcomes of different utrophin contexts on MyHC-emb levels, we analyzed 7-week-old QUAD from wt, *mdx*, utrophin over-expressing *mdx*-Fiona transgenic and dystrophin/utrophin-deficient double knockout (*dko*) mice. *Mdx* QUAD presents an inconsistently increased utrophin signal in regenerating fibers (Fig. 5A) and a 2.5-fold increase of total utrophin protein compared to wt muscle (Fig. 5B). The *mdx*-Fiona mouse is an *mdx* transgenic mouse overexpressing the full-length protein, which presents a high utrophin signal uniformly localized at the sarcolemma (Fig. 5A) and a 3-fold increase of total utrophin protein over the *mdx* level (Fig. 4B). Absence of utrophin worsens the dystrophic phenotype (64), and as expected, utrophin was absent in *dko* muscle (Fig. 5A–B).

In 7-week-old *mdx*-Fiona QUAD, co-utrophin/MyHC-emb (Fig. 5A) immunofluorescence revealed a high and uniform utrophin signal at the muscle membrane preventing the need for the regenerative process. Consequently, MyHC-emb positivity, quantified using a co-laminin-a2/MyHC-emb immunofluorescence, showed a highly significant recovery score—99.7% (Fig. 5C, Supplementary Material, Fig. S5B). Overexpression of utrophin also results in a normalization of *myh3* mRNA level with a 99% recovery score (Fig. 5D). In contrast, the absence of utrophin in the dystrophin deficient *dko* mouse results in a higher number of MyHC-emb-positive myofibers (Fig. 5C) and a significantly higher *myh3* mRNA level (Fig. 5D) compared to *mdx* muscle. Thus, there is a positive correlation between regeneration-associated utrophin and MyHC-emb levels in dystrophic muscles, and an inverse correlation between high levels of utrophin at the muscle membrane and MyHC-emb levels in *mdx*-Fiona skeletal muscle.

### Correlation between utrophin, embryonic myosin and muscle function

The percentage of regenerating fibers in muscle biopsies from BMD and DMD patients was previously shown to correlate with the dystrophic clinical severity (55). Fewer positive developmental myosin fibers and reduced regeneration-associated utrophin intensity were noted in the mild dystrophic BMD phenotype, whereas a higher number of positive developmental myosin fibers and increased regeneration-associated utrophin intensity were observed in the more severely affected DMD muscle.

**Figure 6:**
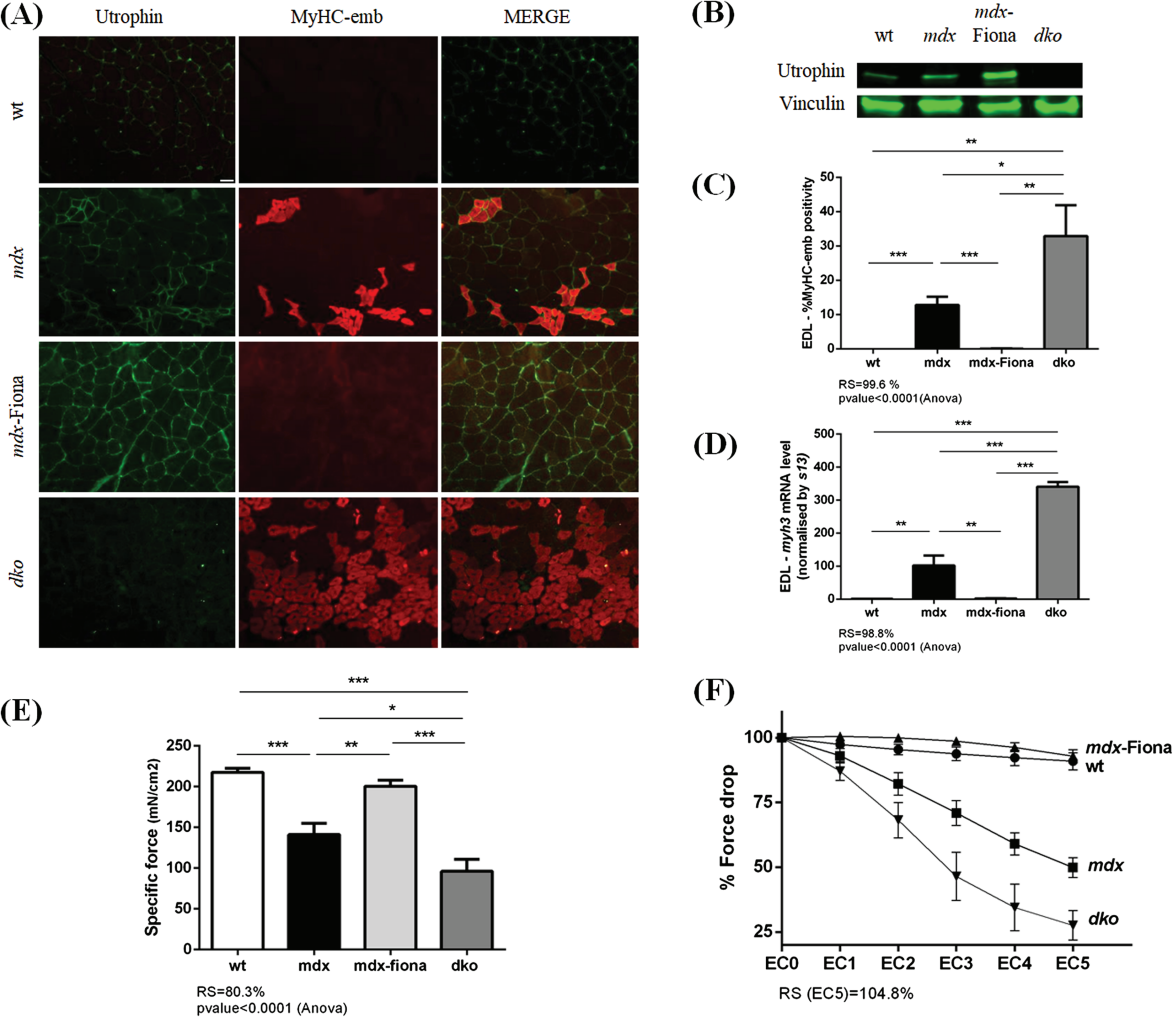
Correlation of utrophin, embryonic myosin and muscle function in 7-week-old EDL muscles. **(A)** Co-immunofluoresence staining for utrophin/MyHC-emb, in 7-week-old wt, *mdx*, *mdx*-Fiona and *dko* EDL. In wt EDL, utrophin is limited to the NMJ and blood vessels and MyHC-emb is negligible. In *mdx* tissue, MyHC-emb signal is increased reflecting a higher regenerative process and utrophin is increased and localized at the muscle membrane of regenerating fibers. In *mdx*-Fiona, utrophin is expressed at high level and consistently localized at the sarcolemma; MyHC-emb is negligible. In *dko*, utrophin is absent, the dystrophic phenotype worsens and MyHC-emb is increased compared to *mdx* tissue. Scale bar: 100 μm. **(B)** Representative relative utrophin protein levels in wt, *mdx*, *mdx*-Fiona and *dko* 7-week-old EDL detected via western blot. Utrophin is increased by 1.7-fold in *mdx* compared to wt; *mdx*-Fiona present a 3-fold increase of total utrophin protein compared to *mdx* and utrophin is absent in *dko*. Vinculin was used to control for equal loading and to normalize utrophin protein expression. **(C)** MyHC-emb positive myofibers in wt, *mdx*, *mdx*-Fiona and *dko* EDL. In *mdx* EDL, 12.7% myofibers are positive for MyHC-emb and high level of utrophin in *mdx*-Fiona fully rescue MyHC-emb to wt levels with a 99.6% recovery score. There is a significant higher number of MyHC-emb positive fibers in *dko* tissue compared to *mdx*. **(D)***myh3* mRNA normalized with *13s* in wt, *mdx*, *mdx*-Fiona and *dko* QUAD. The *myh3* transcripts are fully rescued to wt levels in the utrophin overexpressing *mdx-*Fiona mice. Absence of utrophin in the dystrophin-deficient *dko* results in a significantly higher *myh3* mRNA level compared to *mdx*. **(E)** Specific force in 7-week-old wt, *mdx*, *mdx*-Fiona and *dko* EDL. Dystrophic *mdx* EDL showed a significant reduction in specific force compared to wt; high level of utrophin in *mdx*-Fiona rescues these physiological parameters and absence of utrophin in the dystrophin-deficient *dko* results in a significant decrease of specific force compared to *mdx*. **(F)** Force drop in 7-week-old wt, *mdx*, *mdx*-Fiona and *dko* EDL. Dystrophic EDL muscle shows a progressive and significant decline of force production after damage due to eccentric contractions whereas high levels of utrophin in the *mdx-*Fiona fully prevents this functional decline. Absence of utrophin in the dystrophin-deficient *dko* exacerbates the force drop compared to *mdx*. Values are mean ± SEM of *n* = 6 (wt, *mdx* and *mdx*-Fiona); *n* = 4 (*dko*); ^*^P < 0.05, ^**^P < 0.01, ^***^P < 0.01.

In order to correlate utrophin, MyHC-emb levels and muscle function in mice, we used EDL muscles from 7-week-old wt, *mdx*, *mdx*-Fiona and *dko*. As previously discussed, MyHC-emb levels are significantly increased in *mdx* EDL at this age compared to wt EDL (Fig. 6A, C–D). Regeneration-associated utrophin is increased (Fig. 6A), total utrophin protein content is elevated by 1.7-fold over wt (Fig. 6B), and the specific force (Fig. 6E) as well as the percent force drop (Fig. 6F) are significantly reduced in *mdx* compared to wt EDL muscle.

In *mdx*-Fiona mice, the high and uniform utrophin levels (Fig. 6A–B) rescue the MyHC-emb signal (Fig. 6A–C) and *myh3* mRNA levels (Fig. 6D) toward wt levels. Importantly, muscle function is restored in *mdx*-Fiona and the mice are functionally indistinguishable from wt (Fig. 6E–F). In contrast, compared to *mdx*, the absence of utrophin in *dko* results in a significant increase of MyHC-emb levels (Fig. 6A and C–D) and the muscle physiology is worse with a significant reduction in specific force (Fig. 6E) and in percent force drop after five eccentric contractions (Fig. 6F).

Taken together, our data suggest that utrophin, regenerative processes and muscle function at 7 weeks in EDL muscle are correlated. Increased utrophin related to the regeneration and the increase of MyHC-emb levels in *mdx* skeletal muscle are associated with a reduction of muscle function; absence of utrophin in *dko* triggers a higher regenerative process and worsens the dystrophic pathophysiology whereas the high and uniform increase of utrophin at the muscle membrane in *mdx*-Fiona reduces the regeneration mechanism and rescues muscle function.

## Discussion

In this manuscript, we investigated the expression of MyHC-emb, a well-established marker of regeneration (50) in correlation with dystrophin and utrophin levels in diverse murine skeletal muscles at different ages in order to assess its utility in monitoring muscle health in preclinical studies. In the dystrophic muscles described, recurrent myofiber damage elicits a constant need for skeletal muscle regeneration and the chronic cycles of myofiber necrosis and repair are a hallmark of the disease (1). Whereas developmental myosins precede the appearance of adult fast myosins in the developing skeletal muscle and disappear in most skeletal muscles during early postnatal development (50,51), regenerating muscle fibers in DMD re-express developmental isoforms of myosin as MyHC-emb (52), offering a useful marker of regenerative processes in dystrophic muscles.

In agreement with the pathology of the *mdx* mice characterized by histologically well-defined stages (58), we observed a significant increase of MyHC-emb signal and *myh3* mRNA levels in 7-week-old *mdx* QUAD and EDL skeletal muscle compared to wt muscle. Interestingly, the dystrophic QUAD muscle shows higher MyHC-emb levels than the EDL muscle, highlighting a higher level of regeneration. In line with these results, we quantified a 2.5-fold and 1.7-fold increase of total utrophin protein in *mdx* QUAD and EDL when compared to wt muscle, respectively. As skeletal muscle regeneration is modulated by inflammation (65), this emphasizes that QUAD muscle is more severely affected than the EDL muscle at 7 weeks of age. In 14-week-old *mdx* QUAD, in correlation with the lower regenerative process described in *mdx* muscle (59,60), the reduced but still significantly increased MyHC-emb and *myh3* mRNA were noted. Interestingly at 14 weeks in *mdx* EDL muscle, MyHC-emb levels were not significantly different from wt levels, offering an interesting dystrophic context to study pre-clinical intervention using utrophin modulation. Thus, in agreement with the *mdx* pathology, MyHC-emb is a muscle and age specific marker of regeneration. Importantly, the MYH3 protein cannot be detected by immunoblot after postnatal day 5 in skeletal muscle. Imaging (61) and mRNA quantitation are the only viable solutions to study MyHC-emb.

In order to correlate dystrophin and MyHC-emb levels, we treated 12-week-old male *mdx* mice with Pip-PMO targeting the splicing of exon 23 to restore dystrophin expression. Our results highlight that a short 2-week treatment significantly reduces MyHC-emb levels toward wt, demonstrating a reduction in regeneration. These data suggest that protection of the membrane integrity conferred by dystrophin restoration reduces regeneration in dystrophic muscle. Therefore, increased MyHC-emb levels in DMD patients (52,55,56) may represent a useful therapeutic monitoring biomarker to study regeneration—one of the characteristics of the disease.

We also examined the relationship between MyHC-emb in murine models with differing utrophin levels. We used the transgenic *mdx*-Fiona mice expressing high levels of utrophin and the *dko* model depleted of dystrophin and utrophin. It should be noted that *mdx*-Fiona mice express utrophin under the control of the human skeletal muscle actin promoter from early gestation stages, which prevents pathology. *Mdx*-Fiona mice are histologically and functionally indistinguishable from wt animals (36) and serve as a positive control to show the potential of utrophin modulation strategies. In contrast, *dko* animals lack dystrophin and utrophin during developmental stages resulting in a more severe dystrophic phenotype (64). Our results demonstrate that at 7 weeks of age, in QUAD as well as EDL muscles, overexpression of utrophin fully rescues the MyHC-emb to wt levels and prevents the need to activate the repair process. In contrast, the absence of utrophin significantly increases MyHC-emb levels reflecting a higher regeneration level. Taken together, our data indicate that, similar to dystrophin, utrophin overexpression fully rescues the MyHC-emb levels. Thus, this marker of regeneration could be also used as a monitoring biomarker to study potential benefit of utrophin based strategies.

Recently, a report revealed that the percentage of regenerating fibers correlates with functional motor score in BMD and DMD (55). The authors observed a negative correlation between the level of regeneration and the functional motor score, with a lower motor score associated with an increasing percentage of regenerating fibers in biopsies from DMD patients compared to BMD patients. In limb-girdle muscular dystrophy type 2I, a significant relationship between regeneration, clinical severity and duration of disease was also described, reflecting greater need for muscle regeneration in patients who have lost muscle strength (66). In our study, using different backgrounds of utrophin levels, we noted a correlation between utrophin levels, the regenerative process and muscle function in 7-week-old EDL muscle. The high level of utrophin in *mdx*-Fiona fully rescues MyHC-emb and all functional parameters to wt levels whereas the absence of utrophin results in an increase of embryonic myosin and a dramatic reduction in muscle function.

As part of the repair process, utrophin is increased in dystrophic muscles (31,67). Interestingly, in dystrophic muscle, the regeneration-associated utrophin does not seem protective. Although homogeneous utrophin signal at the sarcolemma across the whole muscle in *mdx*-Fiona mice is highly beneficial, in *mdx* muscle, the regenerative associated utrophin is heterogeneously scattered according to regenerative clusters. Furthermore, regenerative myofibers in *mdx* muscle present a different state associated with different properties compared to the adult utrophin positive fibers in *mdx*-Fiona mice. The turnover and the half-life of the utrophin protein in regenerating *mdx* myofibers could also be different compared to the continuous high level of utrophin observed at the muscle membrane of transgenic *mdx*-Fiona mice. The regeneration-associated utrophin level adds complexity to the overall total utrophin levels and the total utrophin protein content could be misleading in dystrophic muscles without the correlation to regeneration. Recently, a study using semi-quantitative western blots demonstrated similar levels of utrophin protein in two phenotypically discordant DMD half-brothers (68). In addition to the fact that the regenerative process and associated utrophin levels are age-dependent, the level of regeneration across the whole biopsy was not reported in this study and may correlate with disease severity. Distinguishing the initial regeneration-associated utrophin signal in dystrophic muscle from the drug-related utrophin signal is important for the determination of the real impact of a utrophin-based therapy on utrophin level. A robust drug could increase utrophin at the sarcolemma, rescue the membrane stability and subsequently reduce the need for the repair processes and the regeneration-associated utrophin. As MyHC-emb is not quantifiable by western blot after 5 days of life and the aim of utrophin-based strategies is to maintain utrophin in larger non-regenerative muscle fibers, the development of quantitative imaging methods with absolute quantification of utrophin levels at the membrane of each fiber is required (55,69). The key points are the sarcolemmal localization of utrophin in the muscle fibers relative to MyHC-emb and the homogeneity of the utrophin signal across the whole muscle, rather than the absolute utrophin protein level.

Although MyHC-emb is an interesting marker of regeneration for all DMD therapies, notably for utrophin-based strategies, several limitations need to be highlighted. Skeletal muscle regeneration is a highly orchestrated process with sequential but overlapping stages from the inflammatory reaction and the invasion of macrophages: the activation, differentiation and fusion of satellite cells and finally the maturation of newly formed myofibers (70). Even if MyHC-emb signal allows the tracking of the regeneration-associated utrophin signal in dystrophic muscle, there is no single marker that unequivocally identifies a regenerating fiber. Furthermore, MyHC-emb may be present in non-regenerating muscle fibers, such as denervated myofibers (71) and expressed at different levels during the regeneration stages between myofibers. Therefore, MyHC-emb may only offer a partial view of the regenerative process in dystrophic muscle. Thus, complementary indices of regeneration such as fiber size, centrally nucleated fibers, levels of regeneration-associated genes and secreted factors released during muscle repair and which guide muscle regeneration could be informative (60,72). Some skeletal muscle-specific biomarkers such as miR-206, involved in the post-transcriptional activation and repression of utrophin expression (73,74) were also shown to have an essential role during skeletal muscle regeneration (75) and could also serve as an indicator of the regenerative process.

As illustrated in this study, the utrophin signal can be present at the sarcolemma of a small number of non-regenerative myofibers. It was previously shown that utrophin was increased in dystrophic tissue independently from regeneration (76) highlighting that other mechanisms, such as stabilization of the utrophin protein at the muscle membrane, occur in dystrophic muscle.

The data presented in this manuscript describe MyHC-emb as a robust muscle and age-specific marker of regeneration—a hallmark of DMD. We describe the relationship between dystrophin restoration and MyHC-emb as well as the correlation between utrophin, MyHC-emb and muscle function. These data highlight the importance of monitoring utrophin and embryonic myosin levels in preclinical evaluation of utrophin modulators and provide translational support for use of developmental myosin as a disease biomarker in DMD clinical trials.

## Materials and Methods

### Ethics statement

All animal procedures were performed in accordance with UK Home Office regulations, which conform with the European Community Directive published in 1986 (86/609/EEC). The work was performed under certificate of designation number XEC303F12 and project license number 30/3104, following approval by the University of Oxford Department of Physiology, Anatomy & Genetics and Experimental Psychology Joint Departmental Ethics Review Committee.

### Mice

The wt C57BL/10ScSnOlaHsd (C57BL/10), dystrophin-deficient C57BL/1010ScSn-*Dmdmdx*/J (*mdx*), dystrophin-deficient/utrophin-over-expressing C57/Bl10ScSn-*Dmdmdx*/J-Tg (ACTA1-Utrn)2Ked (*mdx*-Fiona) and dystrophin/utrophin *dko* mice were assessed. The C57BL/10 mice were obtained from Envigo (UK) and all other mouse strains were bred in the Biomedical Services Facility, University of Oxford.

### P-PMO synthesis, preparation and administration

Pip9b2 was synthesized by standard solid phase Fmoc chemistry and purified by High performance liquid chromatography, as previously described (62,77). The PMO sequence (5′-GGCCAAACCTCGGCTTACCTGAAAT-3′) was purchased from Gene Tools LLC. Pip9b2 was conjugated to PMO through an amide linkage at the 3′ end of the PMO, followed by purification by HPLC. The final product was analyzed by MALDI-TOF MS and HPLC. For P-PMO arginine-rich cell-penetrating peptide conjugated to a phosphorodiamidate morpholino oligonucleotide treatment, 12-week-old *mdx* mice were administered a single intravenous tail-vein dose of Pip9b2-PMO (10 mg/kg). After 2 weeks of treatment, mice were sacrificed by CO_2_ asphyxiation in accordance with Schedule I of the UK Animals (Scientific Procedures) Act 1986. Muscles were immediately excised and snap frozen in liquid nitrogen or embedded in OCT and frozen in thawing isopentane. Samples were stored at −80**°**C until further analysis.

### Immunofluorescence

Frozen transverse QUAD and EDL muscle sections (10 μm thick) were fixed in acetone for 10 min, washed 5 min in PBS and blocked for 1 h in MOM blocking solution (M.O.M.™ Kit, FMK-2202, Vector Laboratories LTD, Peterborough, United Kingdom). Following 2 × 2 min washes, sections were incubated overnight with primary antibodies at 4°C. The following antibodies and dilutions were used: goat polyclonal anti-utrophin (1:500, URD40), rabbit-polyclonal anti-dystrophin (1:2000; ab15277, Abcam, Cambridge, United Kingdom) and rat monoclonal anti-laminin-α2 (1:50, sc-59854, Santa Cruz Biotechnology). After 2 × 2 min washes and 5 min in MOM diluent (M.O.M.™ Kit, FMK-2202, Vector Laboratories), sections were incubated with a conjugated MYH3 Antibody (F1.652) Alexa Fluor® 594 (1:100; sc-53091 AF594, Santa Cruz Biotechnology, Inc. Dallas, Texas, United States) and appropriate anti-goat (1:2000; A11055, Life Technologies. Carlsbad, California, United States), anti-rabbit (1:2000; A11008, Life Technologies) and anti-rat (1:2000; A11006, Life Technologies) Alexa Fluor® 488 secondary antibodies for 2 h at room temperature. Single staining for F4/80 was performed with the rat anti-mouse F4/80 antibody, clone Cl:A3-1 (1:500, BioRad Laboraties, Hercules, California, United States) and appropriate secondary antibody. Sections were examined under an Axioplan 2 Microscope System (Carl Zeiss, Germany) and multi-acquisition module used to obtain pictures.

### Embryonic myosin quantification

Co-staining Laminin-α2/MyHC-emb were performed on transverse QUAD and EDL sections as previously described and images obtained with the Axioplan 2 Microscope System (Carl Zeiss). Laminin-α2 is used as a mask to obtain total number of muscle fibers per image. Four 10× images per muscle were quantified per muscle and mouse and percent MyHC-emb positivity obtained by dividing the number of muscle fiber positive for MYH3 by total number of fibers. All counting was performed blinded.

### Protein analyses

Muscles samples were homogenized (Polytron 2100; Lucerne, Switzerland) for 3 × 30 s on ice in Radioimmunoprecipitation assay buffer (R0278-50 ml, Sigma-Aldrich) supplemented with protease inhibitors (1:100; P8340, Sigma-Aldrich Company Ltd. Gillingham, United Kingdom). Following bicinchoninic acid assay quantification (23 227, ThermoFisher Scientific, Waltham, Massachusetts, United States), 30–50–100 μg of total protein were heat denatured for 5 min at 100°C before loading onto NuPAGE 3–8% TRIS Acetate Midi Gel (Novex, Life Technologies) and transferred to Polyvinylidene difluoride membranes (Millipore, Burlington, Massachusetts, United States). Membranes were blocked for 1 h with Odyssey Blocking buffer (926-41090, LI-COR; USA) and then incubated with primary antibodies in Odyssey Blocking buffer PBS + 0.1% Tween for 2 h at room temperature. Primary antibodies used were mouse monoclonal anti-utrophin [1:50, MANCHO3(84A), gift from G.E. Morris], rabbit polyclonal anti-dystrophin (1:200; ab15277, Abcam) and mouse monoclonal anti-MYH3 (1:100; sc-53091, Santa Cruz Biotechnology). The Odyssey Imaging System (LI-COR Biosciences, USA) was used to read infrared fluorescence of the secondary antibodies. Relative expression of the target proteins was quantified using vinculin as references and the Image Studio Lite Ver 5.0 software (LI-COR Biosciences).

### RNA analyses

Total RNA was extracted from QUAD and EDL muscle using TRIzol reagent according to the manufacturer’s instructions. A total of 500 ng RNA was used to generate cDNA using the QuantiTect Reverse Transcription kit (205313, Qiagen, Hilden, Germany). Real-time Polymerase chain reaction was performed on the StepOne™ Real-Time Polymerase chain reaction system (Applied Biosystems, Foster City, California, United States) with Fast SYBR™ Green Master Mix (4385612, ThermoFisher). Results were analyzed according to the ΔΔCT method. *myh3* (forward primer 5′-CTTCACCTCTAGCCGGATGGT-3′, reverse primer 5′-AATTGTCAGGAGCCACGAAAAT-3′) and *myh8* (forward primer 5′-CAGGAGCAGGAATGATGCTCTGAG-3′, reverse primer 5′-AGTTCCTCAAACTTTCAGCAGCCAA-3′) mRNA expression levels were normalized to the `normalization factor’ obtained from *13S* Ribonucleic acid (forward prime 5′-CCCGAGGATCTCTACCATT-3′, reverse primer 5′-GCCACTAGACAGAGGCTGT-3′) as reference gene (stability value <1.5). No reverse transcriptase (non-RT), no template control (NTC) reactions and non-contamination of complementary DNA by genomic Deoxyribonucleic acid (ALBh) were used as negative controls in each 40-cycle PCR run (Cq values NTC, undetermined; non-RT, undetermined and ALBh >35).

### Isolated muscle function analysis

Peak force, specific force and force drop were measured from the EDL muscle of the treated and control mice. During dissection and experiments, muscles were bathed in oxygenated (95% O_2_–5% CO_2_) Krebs–Hensley solution composed of (mmol/l): NaCl, 118; NaHCO_3_, 24.8, KCl, 4.75; KH_2_PO_4_, 1.18; MgSO_4_, 1.18; CaCl_2_, 2.54; glucose, 10 (78). Contractile properties were measured as previously described (79). In brief, isolated EDL were attached to a lever arm connected to a force transducer (model 300B) and stimulator (model 701B); the equipment was controlled using the signal interface (model 604A) and results were recorded by the DMC software (version 4.1.4; Aurora Scientific, Aurora, Ontario, Canada). The muscle was stimulated by single pulses of 0.2 milliseconds at 30 V while the optimum length (*L*_o_) was determined. Optimum fiber length (*L*_f_) was calculated by multiplying *L*_o_ by the predetermined fiber length to muscle length ratio of 0.44 (80). A force–frequency curve was generated and the maximum isometric force calculated. Absolute force (*P*_o_) is normalized to specific force (s*P*_o_; mN/cm^2^) using the equation (muscle mass/L_f_ × 1.06) (the density of mammalian muscle). Percentage force drop was calculated by comparing maximum force between the first (ECC0) and fifth eccentric (ECC5) contractions, expressed as a percentage of ECC0. The muscle was stimulated into tetanus at the frequency required to generate the *P*_o_, while in tetanic state the muscle was stretched at a rate of one fiber length per second for 0.15 s, equating to a total stretch of 15% of fiber length. All data were digitized and analyzed using the DMA software (version 3.2, Aurora Scientific).

### Statistics

Results were analyzed using Prism (GraphPad Software, Inc., La Jolla, CA). Comparison of wt, *mdx* and Pip9b2-PMO-treated *mdx*; wt, *mdx*, *mdx*-Fiona and *dko* groups were performed using one-way analysis of variance with post-hoc Tukey test. Student’s *t* test with a two-tailed distribution assuming equal or unequal sample variance depending of the equality of the variance (F-test) was performed to analyze statistical difference between two groups. Recovery score results were obtained using the TREAT-NMD SOP M.1.1_001. Data are presented as mean ± SEM (standard error of the mean), with *n* indicating the number of independent biological replicates used in each group for comparison. Differences were considered significant at (^*^) *P* < 0.05, (^**^) *P* < 0.01 and (^***^) *P* < 0.001.

## Supplementary Material

Supplementary Data
