# Supplementary material for: Embryonic myosin is a regeneration marker to monitor utrophin-based therapies for DMD
Source: Hum Mol Genet. 2018 Oct 9;28(2):307–19. doi: 10.1093/hmg/ddy353 (PMC6322073; doi:10.1093/hmg/ddy353)
Supplement: Supplementary Data [file ddy353_supp.doc]

**Supplementary Materials**

**
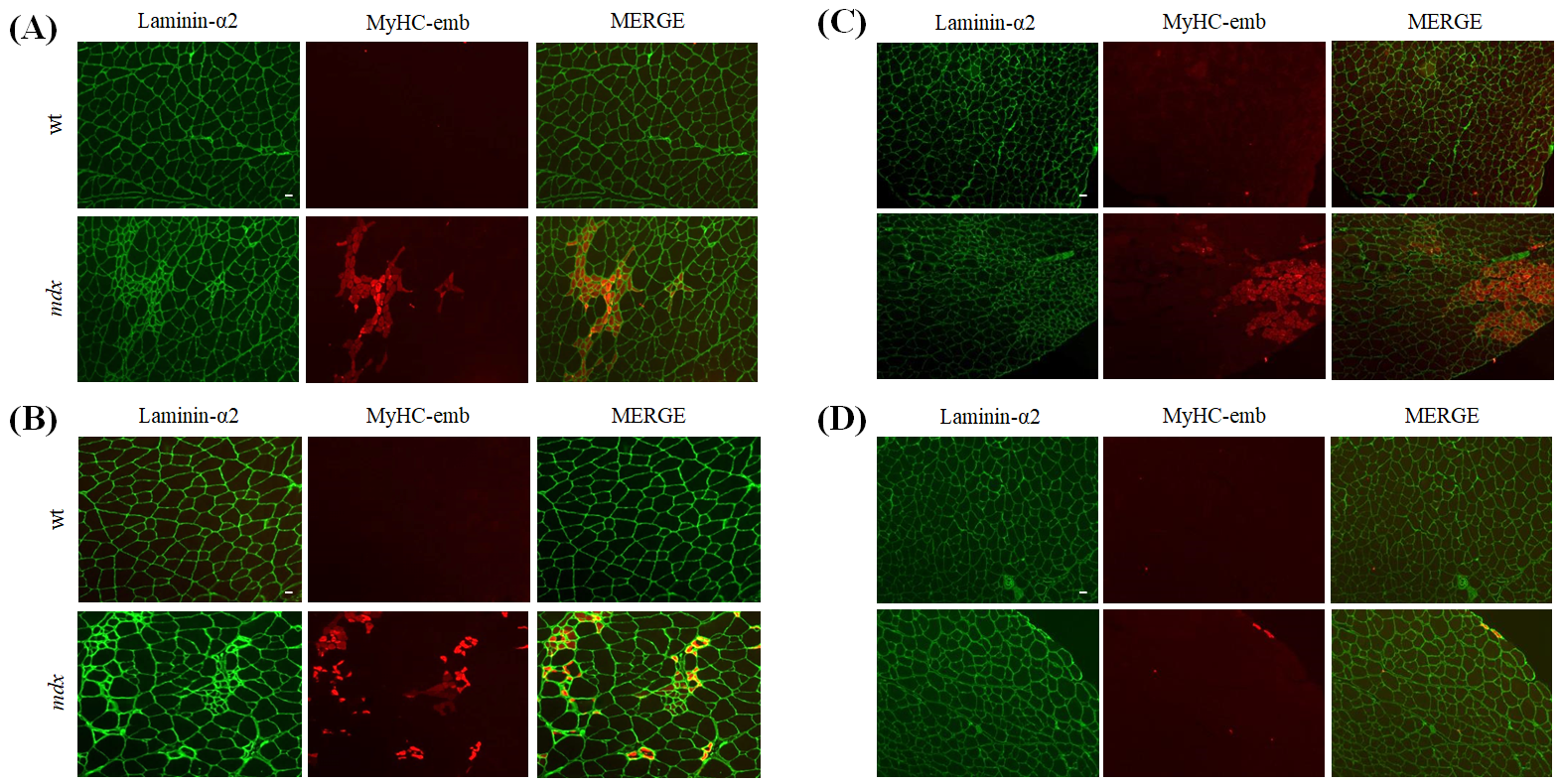
**

**Figure S1: Co-laminin-α2/MyHC-emb immunofluorescence staining in wt and *mdx* skeletal muscles at different ages. (A)** Transverse section of 7-week wt and *mdx* QUAD; (B) Transverse section of 14-week wt and *mdx* QUAD; (C) Transverse section of 7-week wt and *mdx* EDL; (D) Transverse section of 14-week wt and *mdx* EDL. Scale bar: 100µm.

**
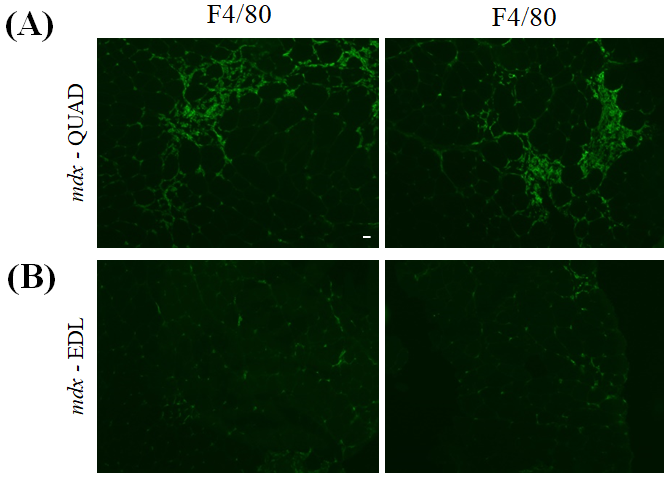
**

**Figure S2: F4/80 staining in 7-week *mdx* QUAD and EDL muscles. (A)** Transverse section of 7-week *mdx* QUAD stained for F4/80, a glycoprotein expressed by murine macrophages. **(B)** Transverse section of 7-week *mdx* EDL stained for F4/80. Scale bar: 100µm.


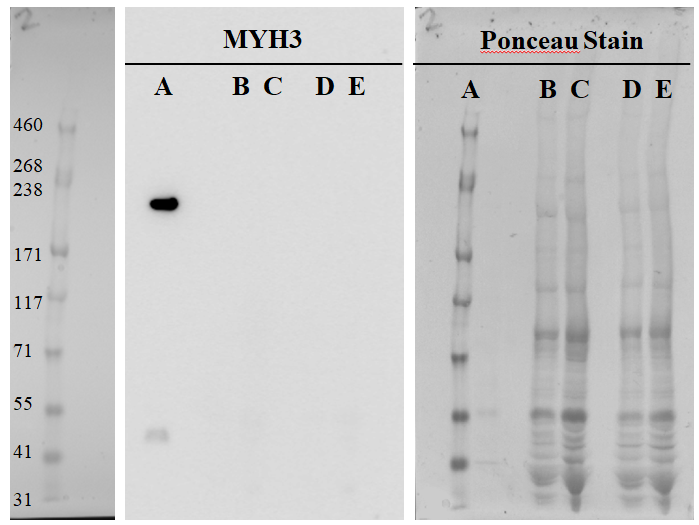


**Figure S3: Relative MYH3 protein levels in 7 and 14-week old wt and *mdx* QUAD detected via western blot.** A: Cardiotoxin treated TA; 5µg Total protein, B: 7wk *mdx* QUAD 50µg, C: 7wk *mdx* QUAD 100µg, D: 14wk *mdx* QUAD 50µg; E: 14wk *mdx* QUAD 100µg.

**
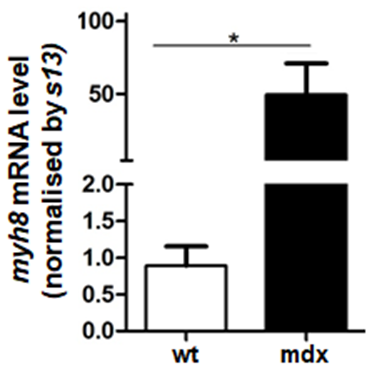
**

**Figure S4: Neonate myosin *myh8* mRNA levels in 7-week old wt and *mdx* QUAD muscles.** *myh8* mRNA normalised with *13s* in 7-weeks old wt and *mdx* QUAD. A significant 53.4-fold increase of the *myh8* transcripts was noted in dystrophic muscle compared to wt. Values are mean ± SEM of n=6 per condition; *p<0.05, **p<0.01, ***p<0.01.

**
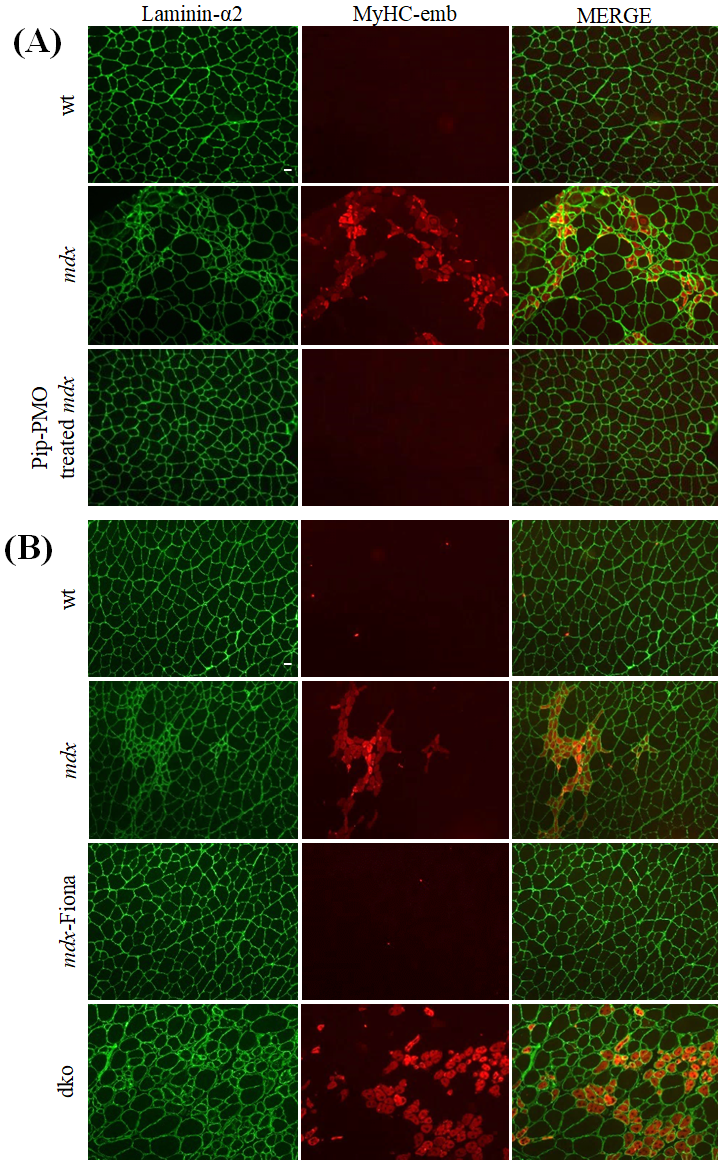
**

**Figure S5: Co-laminin-α2/MyHC-emb immunofluorescence staining. (A)** Transverse section of 14-week wt, untreated and PipPMO treated *mdx* QUAD. Scale bar: 100µm. **(B)** Transverse section of 7-week wt, *mdx*, *mdx*-Fiona and *dko* QUAD. Scale bar: 100µm.
